# Identifying Cyclical Patterns of Behavior Using a Moving-Average, Data-Smoothing Manipulation

**DOI:** 10.3390/bs14121120

**Published:** 2024-11-22

**Authors:** Billie J. Retzlaff, Andrew R. Craig, Todd M. Owen, Brian D. Greer, Alex O’Donnell, Wayne W. Fisher

**Affiliations:** 1 Intermediate School District 917, Rosemount, MN 55068, USA; 2 Golisano Center for Special Needs, SUNY Upstate Medical University, Syracuse, NY 13202, USA; 3 Severe Behavior Department, University of Nebraska Medical Center’s Munroe Meyer Institute, Omaha, NE 68106, USA; 4 Children’s Specialized Hospital–Rutgers University Center for Autism Research, Education, and Services (CSH–RUCARES), Somerset, NJ 08873, USA; 5 Brain Health Institute, Rutgers University, Piscataway, NJ 08854, USA; 6 Robert Wood Johnson Medical School, Rutgers University, New Brunswick, NJ 08901, USA; 7 Department of Psychology, DePaul University, Chicago, IL 60614, USA

**Keywords:** behavior cycles, data smoothing, data analysis, moving average, visual analysis

## Abstract

For some individuals, rates of destructive behavior change in a predictable manner, irrespective of the contingencies programmed. Identifying such cyclical patterns can lead to better prediction of destructive behavior and may allow for the identification of relevant biological processes. However, identifying cyclical patterns of behavior can be difficult when using traditional methods of visual analysis. We describe a data-manipulation method, called data smoothing, in which one averages the data across time points within a specified window (e.g., 3, 5, or 7 days). This approach minimizes variability in the data and can increase the saliency of cyclical behavior patterns. We describe two cases for which we identified cyclical patterns in daily occurrences of destructive behavior, and we demonstrate the importance of analyzing smoothed data across various windows when using this approach. We encourage clinicians to analyze behavioral data in this way when rates vary independently of programmed contingencies and other potentially controlling variables have been ruled out (e.g., behavior variability related to sleep behavior).

## 1. Identifying Cyclical Patterns of Behavior Using a Moving-Average, Data-Smoothing Manipulation

When an individual is referred for the assessment and treatment of destructive behavior, a functional analysis [1] can identify the environmental variables that evoke and maintain the target behavior. When rates of destructive behavior change in a predictable manner, irrespective of the contingencies within the functional analysis, the behavior may be described as cyclical [2], which is a pattern of responding distinct from what is typically expected of automatically reinforced behavior [3]. There are several potential underlying causes of cyclical destructive behavior. For example, caregivers of children diagnosed with autism spectrum disorder who rated their children as poor sleepers also reported more behavior problems compared to caregivers of children diagnosed with autism spectrum disorder who rated their children as good sleepers [4], and escape-maintained destructive behavior may be more likely when the individual has slept less [5,6]. Other researchers have found covariation of destructive behavior with menstrual cycles [7] and have hypothesized that increases in destructive behavior may correlate with increased pain and other physiological changes associated with menstruation [8,9].

Still other case studies have described cyclical destructive behavior in relation to cycle changes experienced by individuals with rapid-cycling bipolar disorder. For example, Lowry and Sovner [10] described two case studies of adults with intellectual disabilities who alternated between depressive and manic states. For one participant, depressive states, which lasted about one week and were characterized by the presence of self-injurious behavior (SIB), alternated with manic states, which lasted about two weeks and were characterized by the absence of SIB. For the other participant, depressive states, which did not include aggression, alternated every few days with manic states, which included aggression. For such individuals, accurate characterization of such cycles by behavior analysts may be instrumental for detecting and treating bipolar disorder through coordinated collaboration with medical professionals.

Fisher et al. [11] described 10 individuals referred for the assessment and treatment of destructive behavior whose behavior appeared to occur in a cyclical pattern. Fisher et al. noted that they were unable to identify a specific underlying cause of cyclical behavior patterns for some of these cases; although, for these cases, the observed pattern was consistent with atypical forms of rapid-cycling bipolar disorder. Additionally, these individuals tended to have poor treatment outcomes. Of the 10 cases described, the authors reported that only one of the individuals showed a meaningful reduction in destructive behavior with reinforcement-based intervention alone. However, eight of the participants showed at least a 70% reduction in destructive behavior with a combination of punishment and differential reinforcement, suggesting that arranging punishment may be necessary to treat most cases of cyclical destructive behavior (to the extent that the authors’ sample was representative of the phenomenon). In another study, SIB in the form of self-slapping was sensitive to water mist as a punisher in an individual with rapid-cycling bipolar disorder [12]. Although there was no effect of water mist during the portion of the cycle associated with a lower rate of SIB (i.e., the trough), SIB was reduced by more than 70% during times of a typical crest (or peak). Finally, although effective treatment of cyclical destructive behavior has been described in the literature, most individuals described by Fisher et al. and Osborne et al. still displayed a cyclical pattern of destructive behavior following treatment, even though the intervention reduced overall rates of destructive behavior [11,12].

The identification of cyclical patterns in destructive behavior can be important for behavior analysts for several reasons. First, the two primary goals of behavior analysis are the prediction and control of behavior [13,14], and identifying cyclical patterns of destructive behavior, when present, can help to predict future changes in behavior. In forecasting research, investigators categorize (a) a cyclical pattern as rises followed by falls in the data that are not fixed in terms of the frequency or duration of the peaks and valleys in the data and (b) a seasonal pattern where they are fixed [15]. For example, destructive behavior that increases and decreases monthly and correlates with a female’s menstrual cycle would be categorized as a seasonal pattern whereas successive peaks and valleys in destructive behavior that vary in duration would be categorized as a cyclical pattern.

Second, caregivers typically seek help, and clinicians typically introduce or change an intervention, when destructive behavior is occurring at high levels. When a treatment is introduced or modified during or near the highpoint of a cyclical pattern, destructive behavior is likely to subsequently decrease, and the caregivers and clinicians may falsely conclude that the intervention produced the improvement in destructive behavior when the reduction was a part of an underlying cyclical pattern. Third, identifying a cyclical pattern in an individual’s destructive behavior could guide and improve treatment evaluations, for example, by extending baseline and treatment phases or by rapidly alternating treatment and comparison conditions in a multielement design over the course of the cyclical pattern [16]. Fourth, the identification of a cyclical pattern should lead the behavior analyst to consider referring the patient for a relevant medical workup (e.g., to a psychiatrist if changes in mood accompany the changes in destructive behavior, to a gynecologist if the changes destructive behavior correlate with a female patient’s menstrual cycle) [11]. In such cases, the behavior analyst may collaborate with the referred physician by providing objective data on the cyclical pattern that may aid the physician with diagnosing the problem or evaluating the effects of a medical intervention.

Given the potential importance of identifying cyclical patterns in destructive behavior when present, and the sparse literature on the assessment or treatment of cyclical destructive behavior, additional research on this topic is sorely needed. However, it can be difficult to study cyclical behavior because when destructive behavior starts to increase, a clinician is likely to adjust the intervention, as mentioned above [11]. Changes in destructive behavior may be falsely attributed to changes in the intervention procedures, and it may take several repetitions of this sequence (i.e., intervention appears to work, intervention stops working, modified intervention appears to work, modified intervention stops working, and so on) before the clinician recognizes an underlying pattern of destructive behavior. In addition, specific data-display or data-aggregation methods are often needed to detect cyclical behavior. For example, data smoothing, a method in which data points are averaged using a moving window, may help to identify cyclical patterns [11]. The purpose of this paper is to demonstrate how data smoothing can aid in the identification of cyclical destructive behavior, which may facilitate the evaluation of effective treatments in children who may not present with a traditional diagnosis indicative of cyclical behavior (e.g., bipolar disorder with rapid cycling).

## 2. Method

### 2.1. Participants and Setting

We included in this analysis data from two participants referred for the assessment and treatment of destructive behavior. Robert, a 9-year-old male diagnosed with oppositional defiant disorder, spoke using full sentences and attended a classroom for children with behavior disorders prior to his admission. Robert had prescriptions for 10 mg of aripiprazole and 20 mg of citalopram in the morning and 18 mg of atomoxetine and 0.1 mg of clonidine in the evening throughout his admission, though he frequently refused to take his medication. Additionally, Robert received 10 mg of *d*-amphetamine in the morning and 0.5 mg of risperidone in the evening at the beginning of his admission, but these medications were discontinued mid-admission. Medication discontinuation had no clear effect on Robert’s destructive behavior. Oliver was an 8-year-old male diagnosed with unspecified disruptive, impulse-control, and conduct disorder, as well as stereotypic movement disorder with self-injurious behavior. Oliver communicated using gestures and by pulling adults toward desired items. He attended an alternate-curriculum classroom prior to his admission and received 10 mg of guanfacine in the morning, 1 mg of clonidine in the evening, and 5 mg of aripiprazole in the morning and in the evening throughout his admission. Oliver rarely refused to take his medication.

Both participants attended an outpatient program that specialized in the assessment and treatment of severe destructive behavior. Appointments for both participants were scheduled 5 days per week for 6 hours per day. We conducted therapy sessions in 3-m by 3-m padded treatment rooms that contained a one-way observation window and two-way auditory communication equipment. Between sessions, participants often transitioned around the clinic, to and from clinic bathrooms, and to and from indoor and outdoor playgrounds.

### 2.2. Response Measurement and Interobserver Agreement

Data collectors recorded frequency data on participants’ destructive behavior using laptop computers during sessions. During clinic transitions and bathroom or playground visits, data collectors recorded frequency data using either hand-held tally counters or paper data sheets. Destructive behavior for each participant included aggression, disruption, and SIB. We operationally defined *aggression* as hitting, slapping, punching, kicking, and biting others; *disruption* as throwing objects, swiping objects off surfaces, overturning furniture, tearing paperwork materials, and kicking or hitting surfaces; and *SIB* as head banging against surfaces, body slamming into walls, self-hitting, self-choking, self-gagging, and self-biting.

An independent second data collector recorded data simultaneously with the primary data collector on participants’ aggression, disruption, and SIB for 24% of Robert’s sessions and 27% of Oliver’s sessions. To calculate interobserver agreement, we divided sessions into 10-s intervals and recorded an agreement for each interval in which observers recorded the same number of responses (i.e., exact agreement within the interval). We then divided the number of agreements within a session by the total number of agreements and disagreements and converted the resultant proportion to a percentage. Mean agreement coefficients for Robert were 99% (range, 76.7–100%), 99% (range, 80–100%), and 98% (range, 80–100%) for aggression, disruption, and SIB, respectively. Mean agreement coefficients for Oliver were 99% (range, 90.9–100%), 99% (range, 91.2–100%), and 99% (range, 85.7–100%), for aggression, disruption, and SIB, respectively. We did not collect interobserver-agreement data on tally-counter or pencil-and-paper data collected outside of session times.

### 2.3. Data Analysis

The primary outcome variable for this study was the daily rate of destructive behavior while in the clinic. Thus, we first added together all instances of destructive behavior that occurred each day for each participant by summing the frequencies of destructive behavior during sessions, clinic transitions, bathroom visits, playground visits, etc. We then divided this sum by the total amount of time participants spent in the clinic that day to convert these frequencies into daily rates of destructive behavior (i.e., responses per hour). Next, we smoothed these data using a moving-average method (see [10], for another example of this approach). We created an Excel file (available in Supplementary Materials) to assist with and automate this step.

The Excel file calculated the moving average based on the inputted daily rates of destructive behavior and a user-defined window of time for analysis. Specifically, the Excel file stepped through the participants’ daily rates of destructive behavior, one day at a time. It then averaged together daily rates of destructive behavior from the current day and from an equal number of days directly preceding and directly following the current day (e.g., a time window of 5 days would include the two days before, the two days after, and the current day). This allowed the total number of data points included in the average to equal the size of the moving-average window. For this reason, we analyzed the data using windows that contained an odd number of days, as an even number of days within a window would require an arbitrary rule of whether to include more days prior to or following the target date. As an example of the approach we used, if the window was 5 days and we wished to calculate the moving-average rate of destructive behavior during Day 10, we averaged together the data from Days 8 through 12. If an insufficient number of days preceded or followed the current day (as was the case at the beginning and end of a dataset), we truncated the number of days included in the moving average. For example, if the window was 5 days and we wished to calculate the moving-average rate of destructive behavior for Day 1, we averaged together the data from Days 1 through 3.

To determine the number of peaks in the data, we surveyed each author as well as five additional responders. Each responder had a minimum of one year’s experience analyzing and interpreting graphical data. Four responders were doctoral-level Board Certified Behavior Analysists (BCBA-Ds), four were BCBAs enrolled in a behavior-analytic Ph.D. program, and two were Registered Behavior Technicians (RBTs). Of note, one of the RBTs had a doctoral degree in behavior analysis and was in the process of completing the experience hours and coursework needed to obtain the BCBA-D credential. We provided each responder with a printed copy of the smoothed data graphs and asked them to indicate peaks in the data and count the number of peaks they identified. If responders indicated more than one data point (i.e., circled more than one data point), we scored the highest point within that group as the peak. We then calculated the number of days between peaks that 80% or more of the responders identified (i.e., inter-peak intervals). We surveyed clinicians to determine the number of peaks as this practice closely mirrors what occurs in most clinical settings (i.e., clinicians visually inspect data).

### 2.4. Behavioral Programming

Throughout the study, both Robert and Oliver received behavioral programming under the supervision of a BCBA-D. This programming included a functional analysis, functional communication training and other reinforcement-based interventions, as well as the use of emergency (Oliver and Robert) and programmatic (Oliver only) restraints. Although a complete description of all analyses and treatment evaluations for both participants is well outside the purview of this study, we included in the sections below a brief overview of our findings with each participant.

Robert’s functional analysis suggested that access to tangible items, escape from demands, and attention in the form of directing adult behavior reinforced his destructive behavior. Robert’s final treatment procedures included a fixed-time alternation between periods of work and periods of reinforcement. The work period was always the first two-thirds of the session, and the reinforcement period was always the final third of the session. However, if Robert completed the work requirement prior to the fixed-time period expiring, his reinforcement period increased accordingly. For example, we designated the first 10 min of a 15-min session as the work period and the last 5 min as the reinforcement period. If Robert completed the assigned work (e.g., one math worksheet) 4 min into the session, this resulted in a reinforcement period lasting 11 min instead of 5 min. Robert was able to access specific, highly preferred reinforcers (e.g., access to the playground) only if he had at least 10 min of reinforcement remaining. We did this to encourage him to complete his work in a quick and efficient manner. Additionally, Robert earned a token for each session he completed without engaging in destructive behavior. Robert later exchanged these tokens for various reinforcers that were unavailable during the reinforcement period within session (e.g., a highly preferred snack). In addition to these procedures, we used an emergency locked-elbow restraint with Robert following SIB that was likely to produce soft-tissue damage (e.g., self-biting).

Oliver’s functional analysis suggested that access to attention and preferred tangible items reinforced his destructive behavior. His final treatment procedures included a multiple schedule in which we alternated between a signaled, 60-s reinforcement component and a signaled, 240-s extinction component. During the reinforcement component, Oliver used a functional communication card to request access to attention and a highly preferred tangible item. During the extinction component, the therapist either delivered instructions using three-step guided compliance, or Oliver had access to a low-preference tangible item. Destructive behavior resulted in a 30-s locked-elbow restraint.

## 3. Results

**Figure 1:**
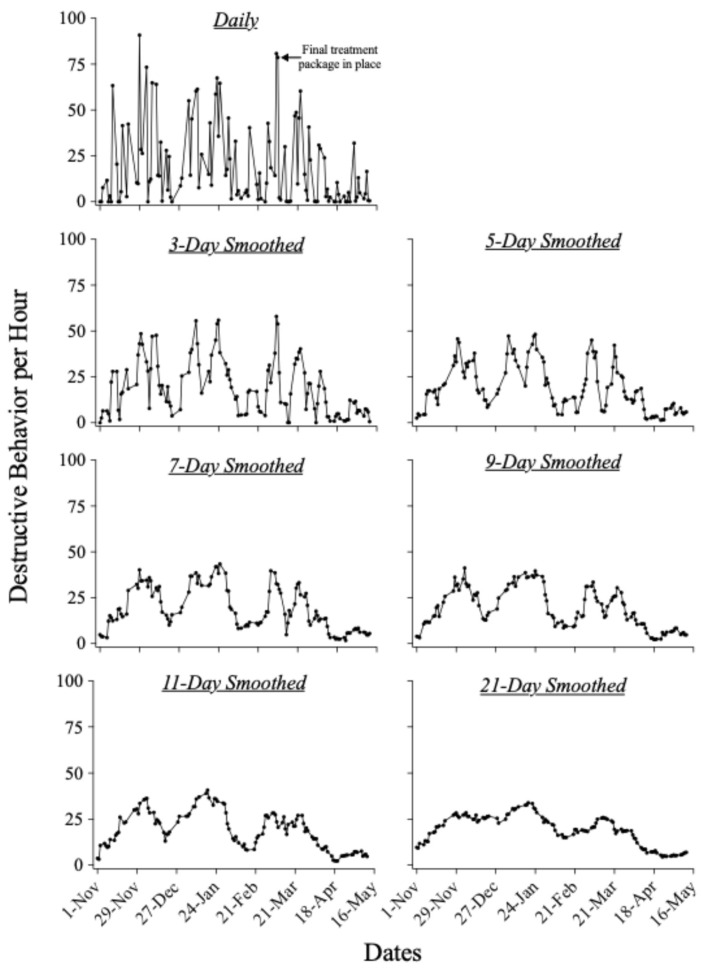
Rate of destructive behavior for Robert.

Figure 1 displays Robert’s daily rates of destructive behavior and these same data smoothed using 3-, 5-, 7-, 9-, 11-, and 21-day moving-average windows. Destructive behavior occurred at variable rates across days when we plotted Robert’s behavior without averaging. Although there appeared to be several distinct increases and decreases in response rate, the highly variable nature of responding across days precluded our assessment of a clear underlying pattern across days. When we smoothed Robert’s data using a moving-average window of 3 days (see the panel titled “3-Day Smoothed”), three distinct periods of elevated behavior began to appear. Substantial increases in Robert’s destructive behavior occurred in late November, early January, and early March with decreases in the rate of destructive behavior between these time points. This cyclical pattern was visible across nearly all moving-average windows. Approximately four weeks separated the peaks, with periods of low-rate destructive behavior separated by roughly the same amount of time. When we smoothed Robert’s data using a 5-day moving average, the cyclical pattern in Robert’s destructive behavior became more discernable, and a pattern of two subpeaks appeared within each period of elevated responding. Smoothing data using a 7-day moving average produced a pattern that was nearly identical to that of the 5-day moving average; however, the double-peak pattern around late November was less visible. After increasing the moving-average window to 9 days, the double-peak pattern across both the first and second periods of increased destructive behavior (i.e., late November, early January) were reduced. Using the 11-day window reduced all three of the double-peak patterns, but the cyclical pattern of increases and decreases in destructive behavior remained. After we increased the window to 21 days, the overall cyclical pattern remained present but was less discernable.

**Figure 2:**
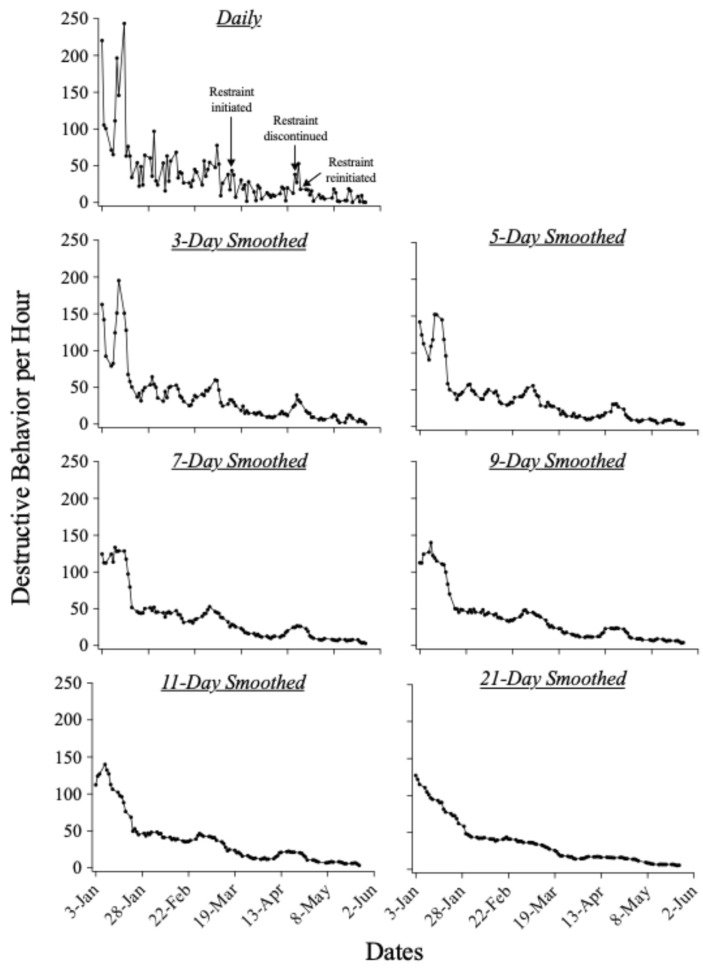
Rate of destructive behavior for Oliver.

Figure 2 displays Oliver’s daily rates of destructive behavior and these same data smoothed with all moving-average windows. As in Robert’s case, rates of Oliver’s destructive behavior without any smoothing method applied (see the panel labeled “Daily”) did not show clear evidence of a cyclical trend in rates of destructive behavior. Overall, rates of destructive behavior tended to decrease across days. Our visual analysis of these data identified periods of high-rate destructive behavior abutted by periods of low-rate behavior, but these periods appeared to occur erratically. When we smoothed these data using 3- and 5-day moving averages, discernable increases in rates of destructive behavior appeared approximately every two weeks, which were followed by abrupt decreases in response rate.

Oliver’s final intervention, which included the use of a locked-elbow restraint for destructive behavior, began in mid-March. This occurred at the beginning of an upward trend in rates of destructive behavior, following which time, the amplitude of subsequent peaks reduced in the 3- and 5-day analyses. Moreover, the locked-elbow restraint seemed to eliminate the cycling pattern in Oliver’s daily rates of destructive behavior, as implementation of the locked-elbow restraint coincided with gradual reductions in response rate during times that otherwise would likely have been characterized by further cycling. Oliver transitioned to a school placement in mid-April, at which time we removed the locked-elbow restraint to comply with school policy. A sizable increase in Oliver’s rate of destructive behavior emerged following removal of the locked-elbow restraint, which was visible across all moving-average analyses except the 21-day averaging window. In collaboration with the school, we reintroduced the locked-elbow restraint several days after he transitioned to school placement and observed a subsequent decrease in rates of destructive behavior. The 3- and 5-day analyses show two small but visible peaks during his 5-week transition to school. Like Robert’s data, the larger moving-average windows produced patterns in Oliver’s data that were less visually discernable and were, therefore, generally of less use to clinicians in a practical sense. Only two major peaks were visible when we used 9- and 11-day averages, and there was no evidence of a continuing cycle in the last month of Oliver’s admission with these larger windows. In the 21-day analysis, there was no clear trend beyond a steady decrease in rates of destructive behavior across days.

To summarize, there was visual evidence of molar trends in Robert’s data across all moving-average windows. The pattern that emerged when we applied our smallest moving-average window (i.e., 3 days) persisted throughout subsequent analyses that used larger windows, though the distinctness of the cycles decreased as the moving-average window increased, as did the number of peaks we identified. In our analyses of Oliver’s destructive behavior, relatively small moving-average windows (i.e., 3- and 5-day windows) revealed regular cycles in destructive behavior. In contrast, the larger averaging windows masked these cycles.

**Figure 3:**
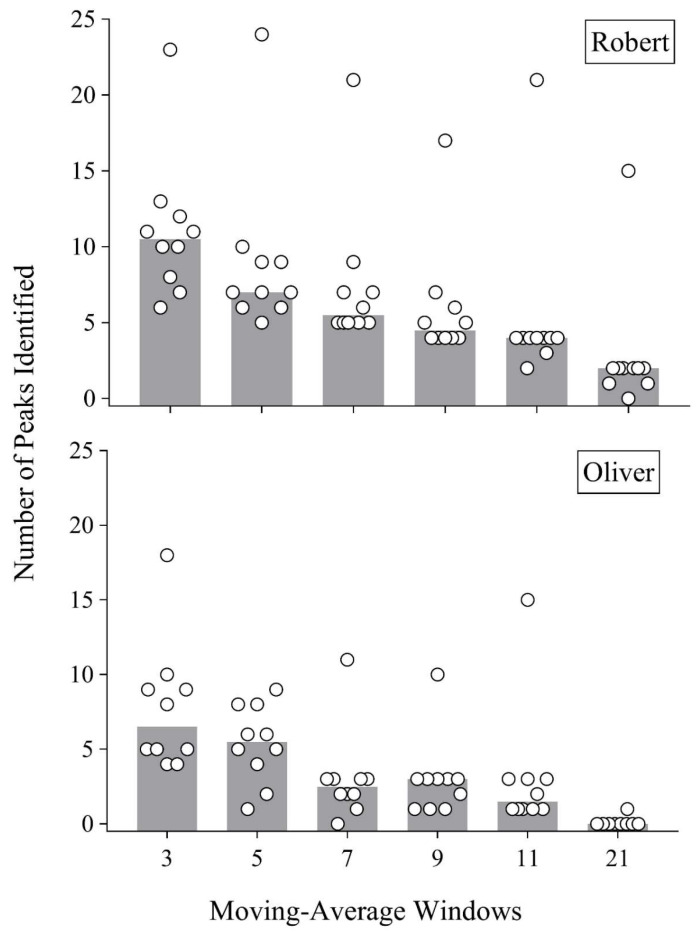
Number of peaks for Robert and Oliver. *Note.* Open circles depict the number of peaks identified by responders at each moving-average window. Gray bars depict the median of those responses.

Figure 3 displays the number of identified peaks in the data for all responders (open circles) and the median of responses across responders (gray bars). As the window size increased, the number of identified peaks decreased for all responders but did so in a slightly more staggered fashion when responders analyzed Oliver’s data. At the 21-day window, most responders continued to identify peaks in Robert’s data, whereas only one responder identified a peak in Oliver’s data with this same window.

**Figure 4:**
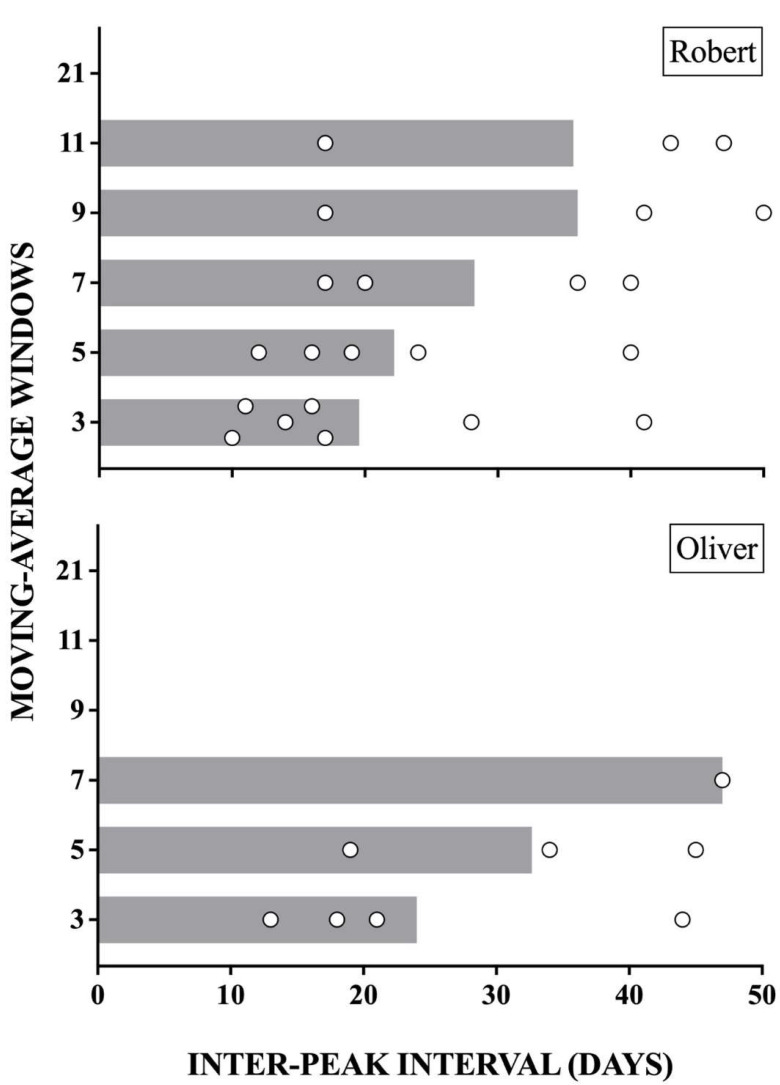
Inter-peak intervals for Robert and Oliver. *Note.* Open circles depict the number of days between peaks (inter-peak intervals) and bars display the median inter-peak intervals.

Responders also provided information regarding the number of days between identified peaks. Figure 4 displays the duration of inter-peak intervals (open circles) as well as the median inter-peak interval (gray bars) for each moving-average window. As the window increased and the number of peaks decreased, the inter-peak intervals increased and then sharply decreased for both participants. Larger windows disproportionally impacted responders’ ability to identify inter-peak intervals. Like the data in Figure 3, the larger windows affected Oliver’s inter-peak intervals more so than they did Robert’s data.

## 4. Discussion

We used the data-smoothing method described by Fisher et al. [11] to illuminate the presence of a cyclical pattern of destructive behavior for two young boys. For both participants, we did not identify the cyclical nature of their destructive behavior via visual analysis when we graphed the daily rate of destructive behavior. However, when we smoothed the data using even a small window, the cyclical nature became apparent. For Robert, we were able to detect the cyclical nature of his destructive behavior with most window sizes; however, once we extended the window to 21 days, the pattern was no longer apparent. For Oliver, when we extended beyond a 5-day window, the cyclical nature of his behavior was difficult to detect. These data suggest that although both participants’ destructive behavior followed a cyclical pattern, the length of the cycle was different for each participant. In addition to the length of the cycle differing, Robert’s rate of destructive behavior tended to follow a two-peak pattern (i.e., an increase in destructive behavior followed by a brief decrease and then an immediate second increase), whereas Oliver’s response rates followed a more uniform pattern. Regardless of the specific pattern of destructive behavior, the data-smoothing method described by Fisher et al. [11] was sufficient to detect the cyclical pattern of behavior. To our knowledge, this is the first application of differently sized moving averages to facilitate interpretation of behavioral phenomena.

We produced clinically significant reductions in rates of destructive behavior with both participants using their final treatment procedures. However, it is important to note that when destructive behavior occurred with the final treatment in place, the cyclical pattern was still apparent, albeit with a reduced amplitude for both participants. Additionally, reinforcement-based procedures alone were ineffective for both participants. Each final treatment included a contingent locked-elbow restraint for destructive behavior (Oliver) or for severe SIB (Robert). These findings align with those reported in the literature on cyclical destructive behavior [11,12] and further suggest that cycles may continue even with effective treatment and that reinforcement-based procedures alone are often insufficient at reducing such behavior.

Our field would benefit from continued research on the prevalence of, and effective treatments for, cyclical behavior. The data-smoothing method described by Fisher et al. [11], and used in the current study, appears to be one effective method for aiding researchers and clinicians in identifying cyclical patterns of responding. Kazdin [17] described a similar method for clarifying patterns in data which involves plotting data as blocks of time. However, the method described by Kazdin is slightly different because a moving window is not used. Instead, data are simply averaged across consecutive blocks of sessions. Using this method, the number of data points included is reduced proportionally to the number of sessions included in each block. This reduction reduces the power of visual and statistical analyses. Therefore, when increased power is advantageous, we recommend using the data-smoothing method described by Fisher et al. [11].

It is important to note several limitations of the current investigation. Although we calculated interobserver agreement for sessions with each participant, we did not have the ability to calculate interobserver agreement for destructive behavior that occurred outside of sessions. Finally, because caregivers administered medications, we did not have complete data for medication adherence for each participant. Thus, although we can report that Robert frequently refused medication and Oliver rarely did, we cannot provide exact information regarding medication adherence. Other variables that may have contributed to the cyclical nature of the participants’ behavior, such as changes in sleep, were also not available in full for analysis in conjunction with rates of destructive behavior.

Clinicians ought to consider using a data-smoothing method only after considering other sources of behavioral variability. If the clinician suspects cyclical destructive behavior, we recommend minimizing changes to the treatment protocol (to the degree feasible while maintaining safety) to increase the likelihood of detecting a cyclical pattern. Once detected, we recommend the clinician consider the duration of the cycle for the individual prior to introducing treatment to ensure that underlying cyclical patterns of behavior do not interfere with an accurate interpretation of treatment efficacy. We recommend first evaluating reinforcement-based interventions while continuing to monitor the cyclical pattern. If reinforcement-based procedures prove ineffective, the inclusion of punishment may be unavoidable, as suggested by the available literature. Even then, some cycling may occur.

## Data Availability

The data presented in this study are available upon request from the corresponding author to ensure participant privacy.

## Supplementary Materials

The following supporting information can be downloaded at: https://www.mdpi.com/article/10.3390/bs14121120/s1, File S1: Data Smoothing Workbook.
